# Molecular docking, molecular dynamics simulations and *in vitro* screening reveal cefixime and ceftriaxone as GSK3β covalent inhibitors†

**DOI:** 10.1039/d3ra01145c

**Published:** 2023-04-11

**Authors:** Husam Nassar, Wolfgang Sippl, Rana Abu Dahab, Mutasem Taha

**Affiliations:** a Department of Medicinal Chemistry, Institute of Pharmacy, Martin-Luther University Halle-Wittenberg Halle (Saale) 06120 Germany; b Department of Clinical Pharmacy and Biopharmaceutics, School of Pharmacy, University of Jordan Amman 11942 Jordan; c Department of Pharmaceutical Sciences, School of Pharmacy, University of Jordan Amman 11942 Jordan mutasem@ju.edu.jo

## Abstract

GSK3β is a serine/threonine kinase that has been suggested as a putative drug target for several diseases. Recent studies have reported the beneficial effects of cephalosporin antibiotics in cancer and Alzheimer's disease, implying potential inhibition of GSK3β. To investigate this mechanism, four cephalosporins, namely, cefixime, ceftriaxone, cephalexin and cefadroxil were docked into the GSK3β binding pocket. The third-generation cephalosporins, cefixime and ceftriaxone, exhibited the best docking scores due to the exclusive hydrogen bonding between their aminothiazole group and hinge residues of GSK3β. The stability of top-ranked poses and the possibility of covalent bond formation between the carbonyl carbon of the β-lactam ring and the nucleophilic thiol of Cys-199 were evaluated by molecular dynamics simulations and covalent docking. Finally, the *in vitro* inhibitory activities of the four cephalosporins were measured against GSK3β with and without preincubation. In agreement with the results of molecular docking, cefixime and ceftriaxone exhibited the best inhibitory activities with IC_50_ values of 2.55 μM and 7.35 μM, respectively. After 60 minutes preincubation with GSK3β, the IC_50_ values decreased to 0.55 μM for cefixime and 0.78 μM for ceftriaxone, supporting a covalent bond formation as suggested by molecular dynamics simulations and covalent docking. In conclusion, the third-generation cephalosporins are reported herein as GSK3β covalent inhibitors, offering insight into the mechanism behind their benefits in cancer and Alzheimer's disease.

## Abstract 2

Cefixime and ceftriaxone covalently inhibit GSK3β.

## Graphical abstract

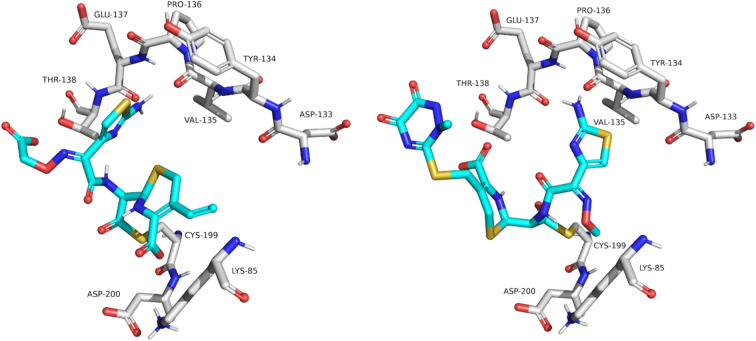

## 1. Introduction

Glycogen synthase kinase 3β (GSK3β) is a ubiquitously expressed serine/threonine protein kinase involved in the regulation of many key cellular processes.^1^ This multifunctional enzyme was initially linked to type II diabetes as one of the kinases that modulate glycogen synthase through phosphorylation leading to its inhibition and, consequently, elevated serum glucose level.^2^ Subsequently, it was reported to be implicated in cancer through inhibition of apoptotic pathways and in Alzheimer's disease (AD) through influencing tau hyperphosphorylation.^3–7^ Accordingly, inhibition of disrupted GSK3β signalling is highlighted as an attractive therapeutic approach for a wide spectrum of diseases.^8–10^

Recently, a plethora of GSK3β inhibitors displaying good-to-excellent *in vitro* activities have been discovered, most of which are ATP-competitive inhibitors.^11^ The development of such inhibitors against GSK3β is enhanced by our understanding of the structural features of its ATP binding pocket and the binding interactions of potent inhibitors. All ATP-competitive inhibitors establish hydrogen bonds with the carbonyl oxygen and/or peptidic amine of Val-135 in the hinge region. Hydrogen bond with the carbonyl oxygen of Pro-136 also appears in several GSK3β-inhibitor complexes. Moreover, hydrogen bond formations with Asp-200 in the DFG motif and the catalytic Lys-85 represent two important interactions for higher activity.^12^ Adjacent to the DFG motif, the nucleophilic thiol of Cys-199 offers an advantage for the development of irreversible GSK3β inhibitors harbouring electrophilic warheads.^13^ On the other hand, GSK3β has a flat and elongated pocket along the hinge region making it more accessible to orthogonal binding orientations of its inhibitors.^14^ GSK3β is also known to exhibit significant anisotropic motion of its active site residues, indicating the GSK3β binding pocket to be induced to fit different ligand binding modes.^15^

**Fig. 1:**
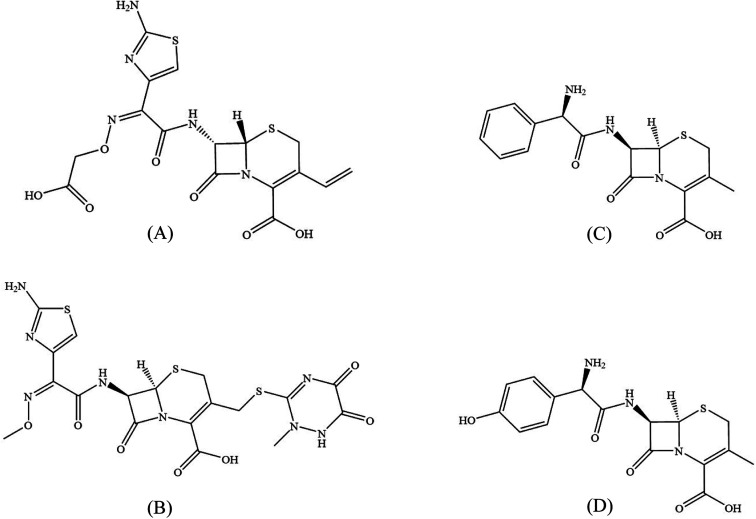
Chemical structures of the four tested cephalosporin antibiotics; cefixime (A), ceftriaxone (B), cephalexin (C) and cefadroxil (D).

Cephalosporin antibiotics are suggested herein to exhibit GSK3β inhibitory activity *in vitro* based on literature reports and molecular docking studies. Cephalosporins are among the most widely prescribed antibiotics because of their broad-spectrum bactericidal activity, verified safety profile and favourable pharmacokinetic properties.^16^ The backbone of cephalosporin antibiotics contains a cephem fragment; a β-lactam ring fused with a six-membered dihydrothiazine ring, to which an amine group is attached at 7-position, forming the 7-aminocephalosporanic acid. Substitutions at positions 3 and 7 led to the discovery of various cephalosporin compounds in clinical use today.^17^ Apart from their bactericidal effect, these β-lactam antibiotics, particularly the third-generation cephalosporins, were reported to interfere with other pathogenic events in cancer and AD. In cancer, cephalosporins were noted to have antiproliferative, cell cycle arresting and apoptosis induction properties.^18,19^ In AD, they were observed to decrease amyloid-β peptide production and upregulate glutamate transporter-1 mediated by the inhibition of tau hyperphosphorylation.^20^ Given the role of GSK3β in these pathophysiologic pathways, the benefits of cephalosporins may be mediated by the inhibition of this kinase.^21^ To test this hypothesis, cefixime and ceftriaxone (third-generation cephalosporins) in addition to cephalexin and cefadroxil (first-generation cephalosporins) were docked against the binding pocket of GSK3β. Cefixime and ceftriaxone showed hydrogen bonding with hinge residues that is essential for ATP-competitive inhibition of GSK3β. Both compounds were further subjected to molecular dynamics (MD) simulations and covalent docking to examine their stability and potential to form a covalent bond with Cys-199 residue. Eventually, our computational studies were validated by *in vitro* bioassay of the four cephalosporin antibiotics (Fig. 1).

## 2. Materials and methods

### 2.1 Protein preparation

Two crystal structures of GSK3β were retrieved from the Protein Data Bank (PDB) and prepared with the protein preparation wizard tool implemented in Schrödinger^21^ (LLC, New York, NY).^22^ GSK3β complexed with the reversible imidazo[1,5-*a*]pyridine-3-carboxamide inhibitor (PDB code: 6Y9S, IC_50_ = 72 nM) was used in Glide SP molecular docking while the second structure complexed with the covalent inhibitor [1,2,4]triazolo[1,5-*a*][1,3,5]triazine (PDB code: 6H0U, IC_50_ = 170 nM) was used in covalent docking.^23,24^ Chain A and the cognate inhibitor were kept in both protein structures. In the case of 6H0U crystal structure, the covalent bond with Cys-199 residue was broken using the Build tool before preparation. Hydrogen atoms and missing side chains were added while ions and water molecules were removed from the X-ray structures. The protonation state and tautomeric forms of the amino acids were optimized using PROPKA tool at pH 7.0.^25^ Finally, the potential energy of the prepared structures was minimized using OPLS4 force-field.^26^

### 2.2 Ligand preparation

The 3D structures of GSK3β cognate inhibitors in addition to the four tested cephalosporins were downloaded as SDF files from the PubChem database.^27^ The compounds were prepared by the LigPrep module in Schrödinger Suite and energy-minimized using OPLS4 force-field.^26,28^ All possible tautomeric forms and stereoisomers were generated at pH 7.0 ± 1.0 using Epik.^29^ Conformations of the prepared compounds were generated using the ConfGen tool by applying 64 conformers per each ligand and minimizing the conformers. All conformers were used as input for molecular docking, whereas the lowest energy conformations were selected for covalent docking.

### 2.3 Molecular docking

The molecular docking study was conducted with the Glide program using the Standard Precision (SP) mode. The grid box was generated around the co-crystalized imidazo[1,5-*a*]pyridine-3-carboxamide ligand (PDB code: 6Y9S) with an inner box of 10 × 10 × 10 Å in size employing the Receptor Grid Generation module. To validate the docking protocol, re-docking of the co-crystallized inhibitor was done and Glide was correctly reproducing its binding mode with RMSD value of 0.57 Å. A maximum of 50 docking poses were calculated for each compound conformer and all other settings were kept as default. The generated poses were scored by glide docking score and glide interaction energy. Ultimately, top-scored poses were analyzed by visual inspection and taken further for MD simulations studies.

### 2.4 Molecular dynamics simulations

The stability of the binding modes of the top-ranked poses obtained after molecular docking was checked by performing MD simulations for 100 ns. The Desmond simulation package was employed to set up the systems and run the MD simulations.^30^ The systems were solvated using the TIP3P water model in a Periodic Boundary Conditions orthorhombic box of 10 Å and neutralized with Cl^−^ ions.^31^ To avoid the interference of Cl^−^ ions in the interactions, placement within a distance of 15 Å from ligand atoms was excluded. For all the simulation runs, the OPLS4 force field and *NPT* (temperature (*T*), pressure (*P*), and the number of particles (*N*)) ensemble was utilized. Prior to performing the production simulation, the default Desmond protocol for energy minimization and model relaxation was applied. A cutoff of 9 Å was used to smoothly truncate the Lennard–Jones interactions and short-range coulombic interactions. The Particle-Mesh Ewald (PME) summation was used to calculate the long-range electrostatic interactions.^32^ Finally, 100 ns MD simulations with a trajectory interval of 200 ps were carried out at a temperature of 300 °K and a pressure of 1.01325 bar in the *NPT* ensemble using a Nose–Hoover chain thermostat and a Martyna–Tobias–Klein barostat.^33,34^ The trajectories were saved at 2 fs intervals for further analysis using Simulation Interaction Diagram (SID) and Simulation Event Analysis (SEA) tools implemented in the Desmond MD package. SID was used to analyse the interactions between the compound and the protein. The stability of the complexes was monitored by examining the Root Mean Square Deviation (RMSD) and Root Mean Square Fluctuation (RMSF) over time using frame zero as a reference. SEA was used to monitor the distance between the β-lactam electrophilic carbonyl carbon and the Cys-199 nucleophilic thiol sulfur. A distance of 3.5 Å was set as a threshold, based on the summation of van der Waals radii of carbon and sulfur atoms, above which covalent bond formation is improbable. To confirm the results of stable poses, MD simulation runs were repeated at a different random seed.

### 2.5 Covalent docking

To inspect the binding poses upon covalent bond formation, covalent docking was carried out employing the CovDock software. CovDock begins with Glide docking to the target with the reactive residue trimmed to alanine. The reactive residue is then added and sampled to form a covalent bond with the ligand in different poses.^35^ Cys-199 was defined as the reactive residue and the docked compounds were confined to an enclosing box marked by the centroid of the workspace [1,2,4]triazolo[1,5-*a*][1,3,5]triazine covalent inhibitor (PDB code: 6H0U). The reaction type of the covalent inhibitor and cephalosporins were chosen as Michael addition and β-lactam addition, respectively. The docking mode was set to pose prediction (thorough). A maximum of 10 poses per ligand were generated and scored according to their cdock_affinity. Complexes showing the highest affinity scores were selected. For the covalent inhibitor, the covalent docking protocol successfully reproduced the co-crystalized binding mode with an RMSD of 1.3 Å.

### 2.6 Kinase inhibition assay

Cefixime, ceftriaxone, cephalexin and cefadroxil were kindly provided as powders by Hikma Pharmaceuticals (Jordan). The compounds were dissolved in 100% (v/v) DMSO and further diluted to obtain the final tested concentration. Enzyme inhibition bioassay was performed using Invitrogen Z'-LYTE® Kinase Assay.^36^ Each cephalosporin was dissolved in 100% (v/v) DMSO to form a 10 mM stock solution (2 mL). The four tested cephalosporins were initially screened at 100 μM against GSK3β. The bioassay was conducted in black 384-well plates. For testing each compound, 100 nL of the stock solution (10 mM) was mixed with 5 μL of the peptide/kinase mixture, 2.4 μL of the kinase buffer (50 mM HEPES pH 7.5, 0.01% BRIJ-35, 10 mM MgCl_2_, 1 mM EGTA) and 2.5 μL of the ATP solution. The final 10 μL kinase reaction included the tested cephalosporin concentration (100 μM), 1% (v/v) DMSO, kinase peptide substrate and 25 μM ATP in 50 mM HEPES pH 7.5, 0.01% BRIJ-35,10 mM MgCl_2_, and 1 mM EGTA. The mixture was shaken for 30 s then incubated at room temperature for 60 min to complete the kinase reaction. Subsequently, 5 μL of the development reagent solution was added to each reaction mixture, shaken for 30 s and left for another 60 min at room temperature. Ultimately, fluorescence emissions were measured at *λ*_ex_ of 445 and *λ*_em_ of 520 nm. Cefixime and ceftriaxone resulted in >60% inhibition and were considered for further screening to calculate their IC_50_ values. The concentrations of 10, 1, 0.1 and 0.01 μM were applied. The tests were performed in duplicates and staurosporine was used as a positive control, tested in the same conditions.^37^ To assess the potential of both compounds to irreversibly inhibit GSK3β, they were initially incubated at 10 μM with the kinase for 60 min before adding the ATP solution. Since covalent bond formation is a slow process compared to reversible attractions, this additional preincubation step allows a productive juxtaposition of the reactive partners as a result of prolonged residence time within the kinase binding pocket. Eventually, the shift in IC_50_ was determined by measuring the percent inhibition at 1, 0.1 and 0.01 μM after applying the same preincubation step.

### 2.7 Data analysis

Docking poses and figures were rendered by using PyMOL (The PyMOL Molecular Graphics System, Version 1.8.4). Statistical analysis of the kinase bioassay results was performed using GraphPad Prism version 7 software. IC_50_ values for cefixime and ceftriaxone were calculated by nonlinear regression analysis. Dose–response curves were formed by plotting the percentage inhibition *versus* log concentration.

## 3. Results

### 3.1 Molecular docking studies

**Fig. 2:**
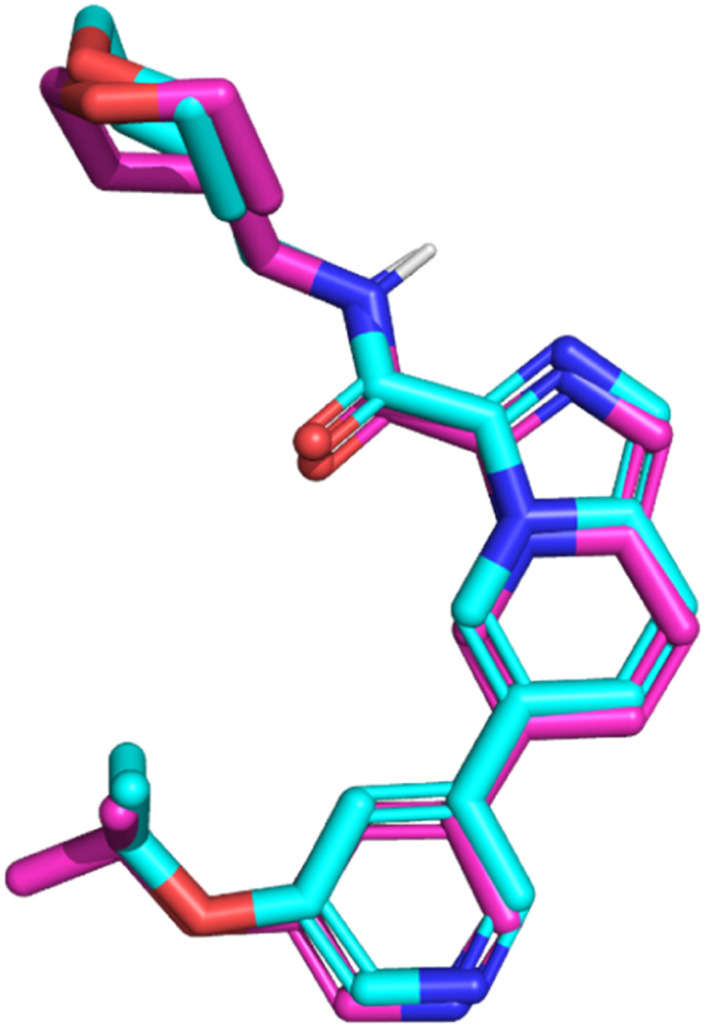
Comparison between the docked pose of the GSK3β inhibitor as produced by the docking study (cyan) and the original crystallographic structure of the same inhibitor within the GSK3β binding pocket (magenta).

**Fig. 3:**
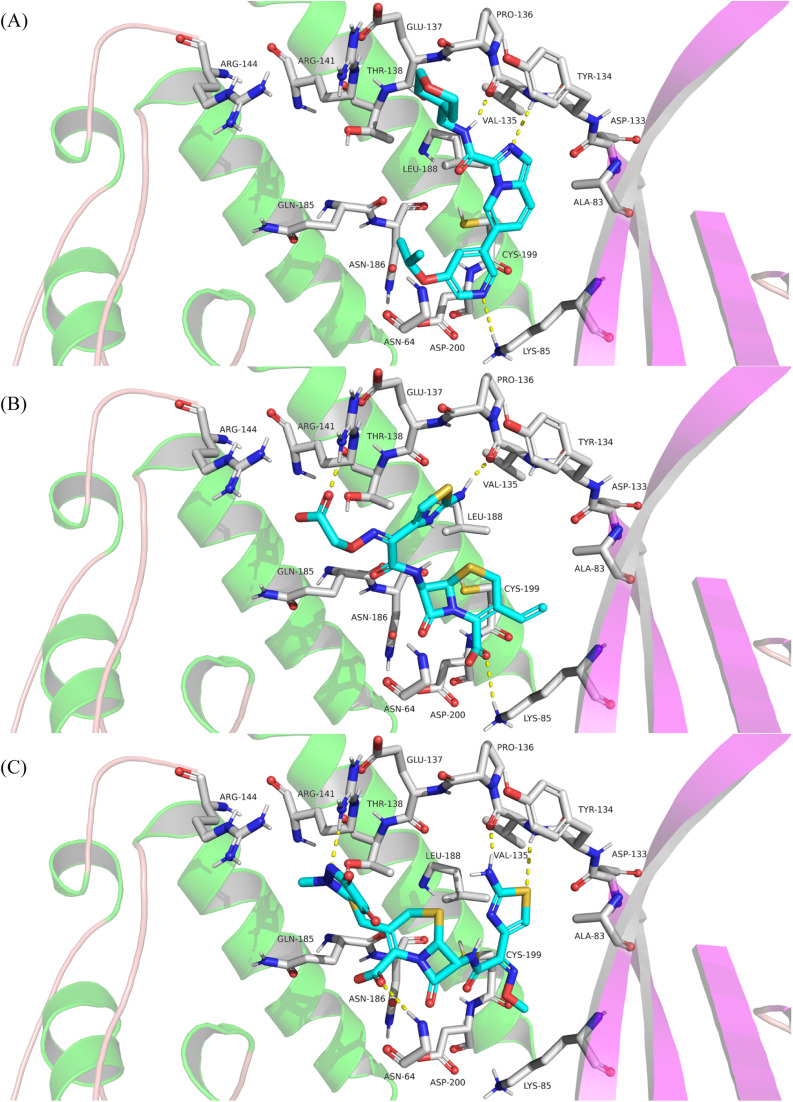
Detailed view showing the interactions of amino acid residues within the binding pocket of GSK3β with the co-crystallized inhibitor (PDB code: 6Y9S) (A), cefixime (B) and ceftriaxone (C). Inhibitors are colored cyan. Hydrogen bonds (distance below 3.0 Å) are shown as yellow dashed lines.

Molecular docking was performed to study the binding interactions of the four cephalosporins within the GSK3β binding pocket using the Glide docking program. The docking protocol was validated based on its ability to reproduce the binding mode of the GSK3β-bound co-crystalized inhibitor (imidazo[1,5-*a*]pyridine-3-carboxamide inhibitor 16 in PDB 6Y9S). This was accomplished by superimposing the crystallographic pose of the ligand with its top-scored docked pose (Fig. 2). The docked pose successfully resembled the crystallographic data of the ligand with an RMSD of 0.57 Å, providing impetus to launch the docking process using the same settings. Fig. 3 illustrates the docked pose of the GSK3β bound inhibitor (PDB code: 6Y9S) compared to the top-ranked poses of cefixime and ceftriaxone. Docking of cephalexin and cefadroxil revealed a common binding mode with the GSK3β binding pocket while interactions with the hinge region were absent confirming the essential role of aminothiazole as the hinge interacting group (ESI Fig. S1†). However, cefixime and ceftriaxone were anchored to the hinge region *via* hydrogen bonding, salt bridges and/or π–cation interaction while exhibiting opposite orthogonal binding orientations (Fig. 3B and C). In particular, the aminothiazole group in both compounds was projected toward the amino acids of the hinge region forming a hydrogen bond with the carbonyl oxygen of Val-135, similar to that formed by the amide NH of the co-crystallized potent inhibitor. The oxygen of the carboxylate attached to the dihydrothiazine ring in cefixime and that of methoxyimine in ceftriaxone were docked at proximity to Lys-85 and Asp-200 as seen with the pyridine nitrogen of the co-crystallized inhibitor. The oxygen of the carboxylate in cefixime and the nitrogen of the dioxotriazine ring in ceftriaxone were in contact with the ε-NH_3_^+^ of Arg-141 near the solvent-exposed region, forming a hydrogen bond and salt bridge, respectively. Additionally, the ε-NH_3_^+^ of Arg-141 was involved in π–cation interaction with thiazole ring of cefixime and triazine ring of ceftriaxone. The vinyl moiety of cefixime was docked within a hydrophobic pouch of Val-70, Ala-83, Leu-132 and Leu-188 suggesting mutual hydrophobic interactions. Ultimately, the β-lactam electrophilic carbonyl carbon of both compounds was docked at proximity to the nucleophilic thiol of Cys-199. Because cephalexin and cefadroxil did not show hinge binding interactions required for GSK3β inhibition, energy estimates calculated using glide indicate both compounds form very weak kinase complexes compared to the co-crystallized potent ligand as well as cefixime and ceftriaxone. Table 1 shows calculated binding energy estimates (enthalpies) and docking scores corresponding to the highest-ranking poses depicted in Fig. 3 and S1.† Clearly from Table 1, cephalexin and cefadroxil scored consistently lower negative values compared to the other tested compounds, highlighting the importance of the aminothiazole group in binding affinity.

**Table 1:** Glide SP docking scores and interaction energies (kcal mol^−1^) calculated for docked GSK3β inhibitor and cephalosporins

Compound	Glide scores		GSK3β inhibition at 100 μM (given in 100%)^a^
	Glide SP docking score	Glide interaction energy	
GSK3β inhibitor (RMSD: 0.57 Å)	−9.92	−58.42	—
Cefixime	−7.36	−48.28	96 ± 1
Ceftriaxone	−7.06	−61.38	67 ± 2
Cephalexin	−6.30	−42.25	37 ± 1
Cefadroxil	−6.19	−42.54	25 ± 1

a Average of duplicate measurements ± SD.

### 3.2 Molecular dynamics simulations

**Fig. 4:**
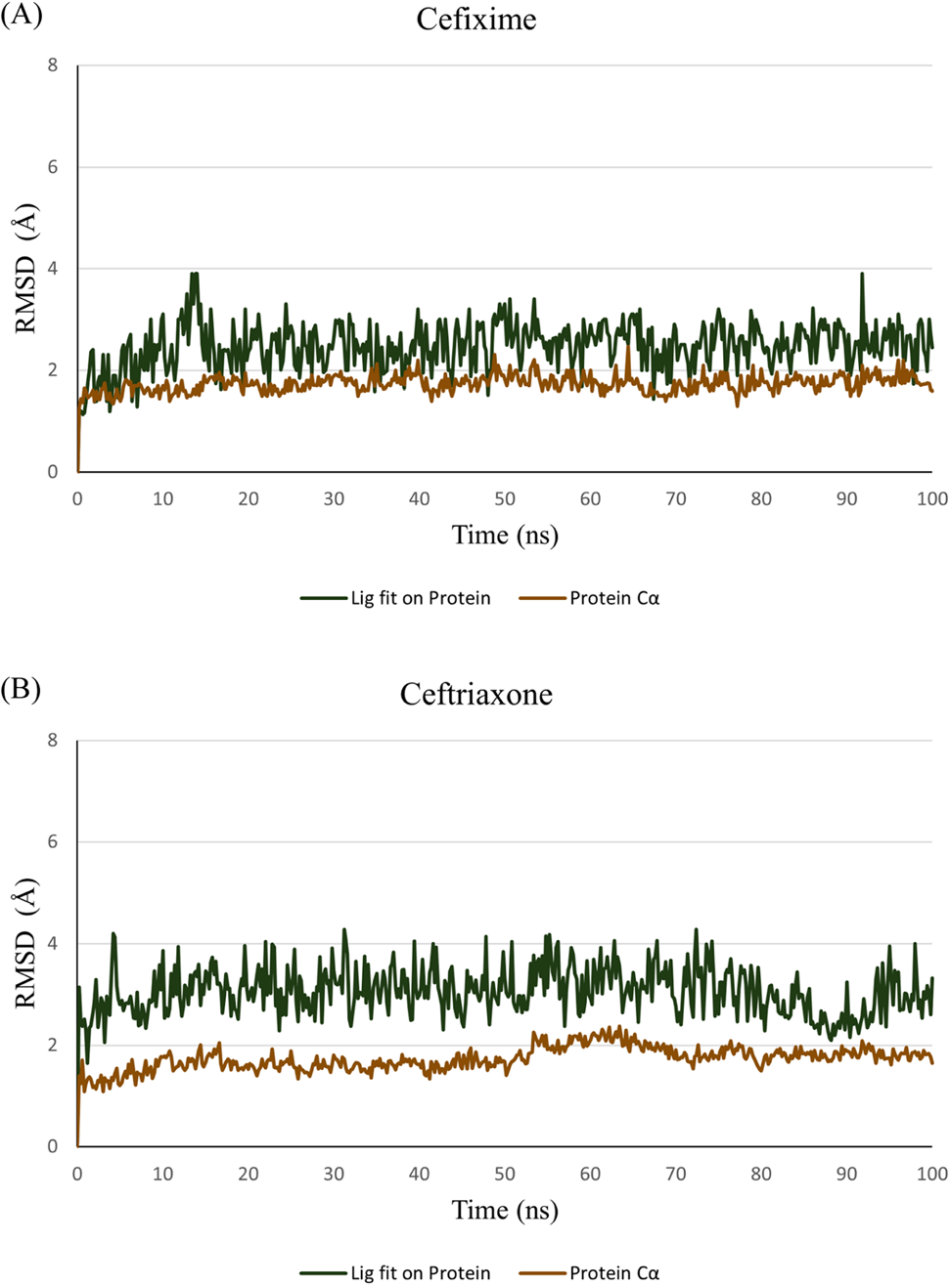
RMSD values throughout 100 ns MD simulations of cefixime (A) and ceftriaxone (B) – GSK3β complexes. RMSD values of protein Cα and docked poses fitting on protein Cα are represented in brown and green, respectively.

To verify the stability of the predicted binding modes obtained by Glide SP docking for cefixime and ceftriaxone, the retrieved poses-complexes were subjected to 100 ns MD simulations using Desmond package. Specifically, we wanted to investigate if the produced opposite orthogonal binding orientations of both compounds were stable during MD simulation. Moreover, we wanted to check if these binding orientations were the most stable as compared to other binding modes. Thus, docking results depicted in Fig. 3 in addition to two other different binding modes of each compound (Fig. S2†) were used as initial coordinates in setting up the MD systems. The comparison between the simulation results was focused primarily on the stability of the binding modes by calculating RMSD and RMSF values plotted in Fig. 4 and ESI Fig. S3.†

**Fig. 5:**
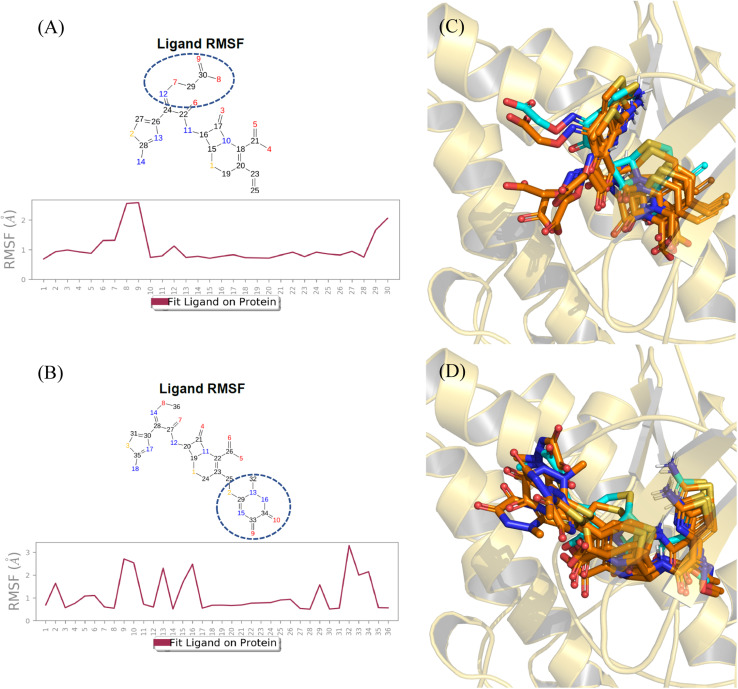
RMSF values retrieved from 100 ns MD simulations of cefixime (A) and ceftriaxone (B) – GSK3β complexes. RMSF values are plotted broken down by atom with the encircled fragments being the most fluctuating parts. In (C) and (D) are shown the binding modes during the simulation at 0 ns, 25 ns, 50 ns, 75 ns and 100 ns (orange sticks) superimposed with the docked poses (cyan sticks).

The analysis of the MD simulation runs of the three different binding orientations of each compound highlighted that the binding modes depicted in Fig. 3 are the most stable. Clearly from Fig. 4, the RMSD of the protein Cα fluctuated between 1.5 and 2.0 Å. The RMSD values of cefixime and ceftriaxone fitting on the protein Cα were within the ranges of 2.0–3.5 Å and 2.0–4.0 Å, respectively, with the initial poses being maintained during the simulation time. The RMSD variations of both compounds are attributable to the solvent-exposed groups (carboxymethoxyimine in cefixime and dioxo-triazine in ceftriaxone) as they are significantly fluctuating outside the binding pocket, showing high RMSF values (Fig. 5). The aminothiazole group of cefixime was moving closer to Pro-136 forming a hydrogen bond with its carbonyl oxygen while the hydrogen bond between the aminothiazole of ceftriaxone and the carbonyl oxygen of Val-135 was preserved. In both compounds, a water-mediated hydrogen bond between the β-lactam carbonyl oxygen and Asp-200 was formed and maintained. Moreover, the catalytic Lys-85 was interacting with dihydrothiazine carboxylate oxygen (in cefixime) and acetamide oxygen (in ceftriaxone) during the whole simulation time. To further confirm the stability of the binding modes and interactions of both poses, the MD simulations were repeated at different random seeds. This helps to ensure that the observed results are robust and not specific to particular positions, velocities and configurations. The RMSD and RMSF values were consistent, confirming the observed stability in comparison to the other binding modes (ESI Fig. S4†). The detailed ligand–protein interactions are described in the ESI (Fig. S5†).

**Fig. 6:**
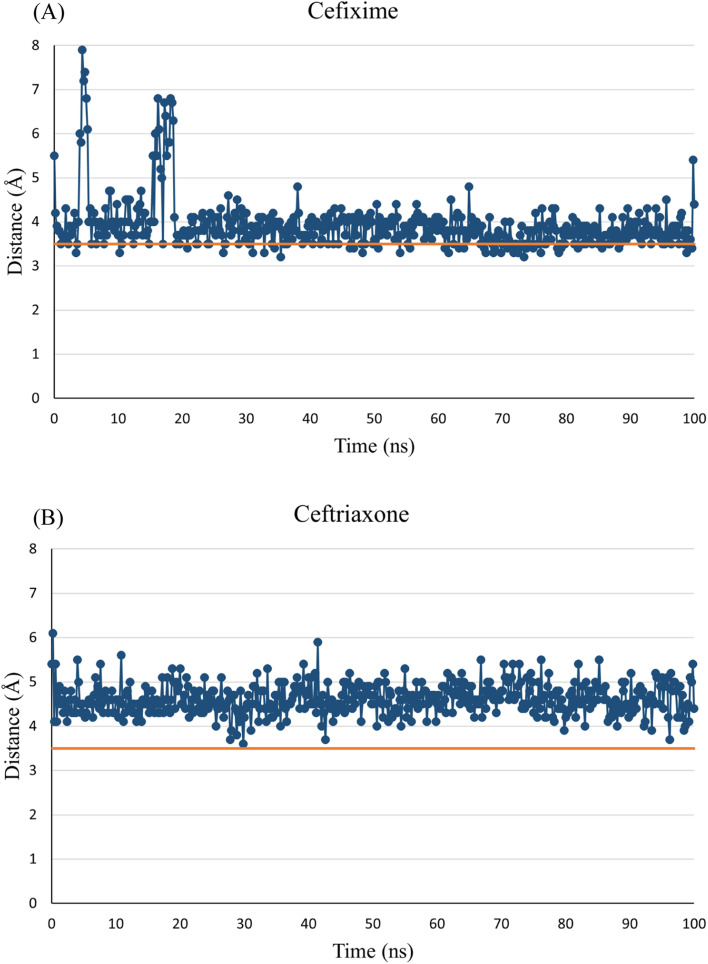
MD simulation distance probes. Distance separating the electrophilic carbon of the β-lactam ring of cefixime (A) and ceftriaxone (B) from the sulfur of Cys-199. The orange line represents the minimum distance between an electrophilic carbon atom and a nucleophilic sulfur atom in a covalent bond productive encounter (this threshold line at 3.5 Å represents the summation of van der Waals radii of carbon and sulfur atoms).

Interestingly, the electrophilic carbonyl carbon of the β-lactam ring in both compounds closely approached the nucleophilic thiol of Cys-199. Accordingly, the distance between the electrophilic carbon and Cys-199 sulfur was monitored during the simulation time. A distance threshold of 3.5 Å (the summation of van der Waals radii of carbon and sulfur atoms) was set, above which the formation of a covalent bond between these two atoms is improbable. Apparently, both compounds hold the potential to form a covalent bond with Cys-199. Cefixime appears to have stronger odds than ceftriaxone as the threshold line was crossed throughout the simulation time whereas, in ceftriaxone, the threshold line was only contacted at some time points (Fig. 6).

### 3.3 Covalent docking

**Fig. 7:**
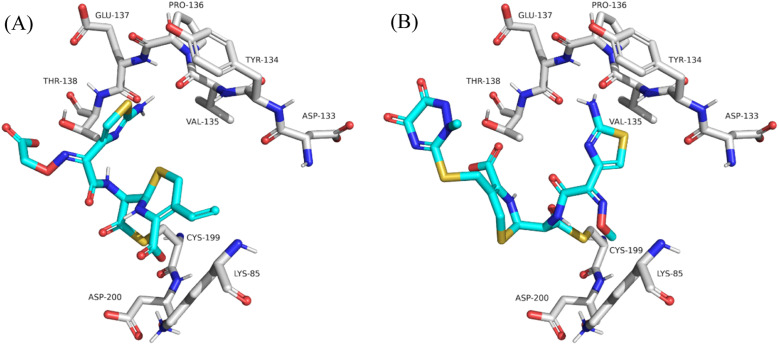
Detailed view showing the predicted binding modes of cefixime (A) and ceftriaxone (B) within the binding pocket of GSK3β upon covalent bond formation.

**Fig. 8:**
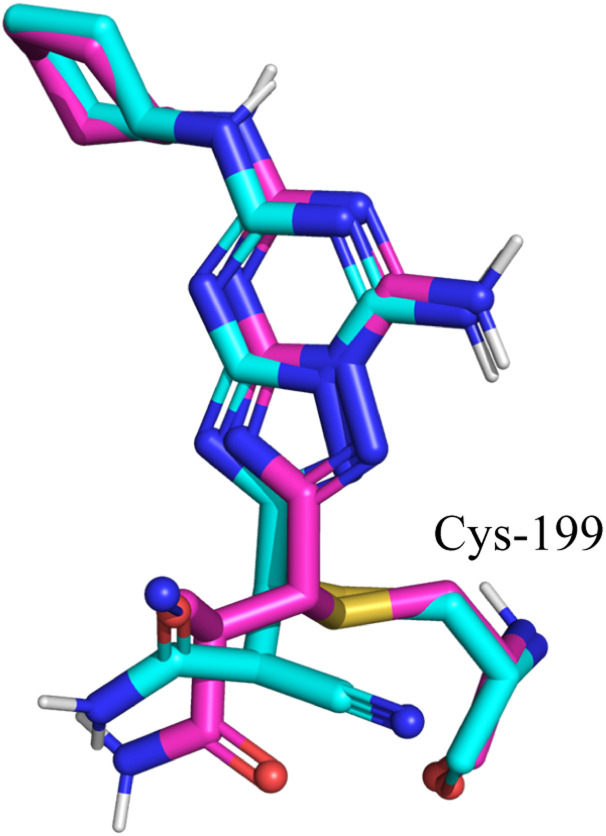
Comparison between the docked pose of the GSK3β inhibitor as produced by the covalent docking (cyan) and the original crystallographic structure of the same inhibitor within the GSK3β binding pocket (magenta).

To inspect the binding mode upon covalent bond formation with Cys-199, cefixime, ceftriaxone, cephalexin and cefadroxil were covalently docked within the GSKβ binding pocket. Covalent docking showed the four compounds to successfully form a covalent bond while the nucleophilic thiol of Cys-199 attacks and ring-opens the β-lactam ring. Consistent with the Glide SP docking, top-ranked poses of cephalexin and cefadroxil failed to interact with the hinge region (Fig. S6†), whereas cefixime and ceftriaxone showed orthogonally opposite orientations with the aminothiazole being projected toward Val-135 and Pro-136 in the hinge region (Fig. 7). Moreover, the carboxylate oxygen of dihydrothiazine in cefixime and the methoxyimine oxygen in ceftriaxone were docked at proximity to Lys-85 and Asp-200. In ceftriaxone, the dihydrothiazine carboxylate was flipped upward to face the hinge region as seen in the MD simulations (Fig. 7B). To compare the stability and interaction patterns of cefixime and ceftriaxone before and after covalent bond formation, an additional MD simulations study of the covalently bound compounds was performed. The results were consistent as the complexes were stable and the interactions with hinge residues (Val-135 and Pro-136), catalytic Lys-85 and Asp-200 in the DFG motif were sustained upon covalent bond formation. The detailed ligand–protein interactions are described in the ESI (Fig. S7 and S8†). Ultimately, the covalent docking protocol was successfully reproducing the [1,2,4]triazolo[1,5-*a*][1,3,5]triazine covalent inhibitor (PDB code: 6H0U) as shown in Fig. 8.

Covalent docking, however, was used as an illustrative tool to predict how cephalosporins fit into the binding pocket in case they succeed in forming a covalent bond. The resulting binding modes nicely correlate with the stable poses produced by Glide SP docking. Nevertheless, we believe the main supportive tool is MD simulations and the observed approaching distance between the electrophilic β-lactam carbon and the nucleophilic Cys-199 thiol.

### 3.4 Cefixime and ceftriaxone inhibit GSK3β *in vitro*

**Fig. 9:**
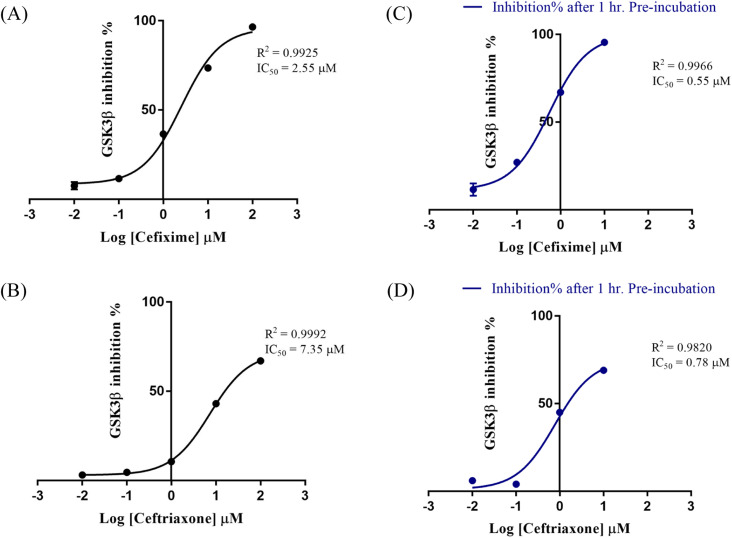
Dose–response curves of the effect of cefixime (A) and ceftriaxone (B) on GSK3β. In (C) and (D) are plotted the % inhibition after 60 min pre-incubation of cefixime and ceftriaxone, respectively. Data are expressed as means of duplicate measurements ± SD.

Encouraged by the above-mentioned results, we screened the four cephalosporin antibiotics against GSK3β using Invitrogen Z'-LYTE® Kinase Assay.^32^ Cephalosporins were initially tested against GSK3β at 100 μM. The percent inhibition results are depicted in Table 1. Consistently, cefixime and ceftriaxone exhibited the best inhibitory profile with a percent inhibition of 67% and 96%, respectively. This prompted us to further evaluate the bioactivities of the two compounds at different concentrations to determine their IC_50_ values against GSK3β. The two compounds resulted in smooth-dose response curves (Fig. 9A and B). Cefixime showed an IC_50_ value of 2.55 μM whereas ceftriaxone exhibited an IC_50_ of 7.35 μM.

To assess the potential of covalent bond formation as suggested by MD simulations, cefixime and ceftriaxone were incubated for additional 60 minutes with GSK3β before measuring the percent inhibition. The activity of irreversible inhibitors is known to be influenced by the residence time and the distance between the reactive electrophile and the nucleophilic trap.^38^ Accordingly, both compounds were initially preincubated with the kinase at 10 μM. The percent inhibition increased by about 30% and was further measured at different concentrations to determine the shift in their IC_50_ values (Fig. 9C and D). Cefixime exhibited an IC_50_ of 0.55 μM while ceftriaxone displayed an IC_50_ of 0.78 μM, supporting the predicted covalent inhibition.

## 4. Discussion

GSK3β is a multi-faceted protein kinase that plays indispensable roles in a diverse range of biological events involved in the regulation of cell fate. Dozens of putative substrates that control glycogen metabolism, protein synthesis and cell apoptosis were characterized to be phosphorylated by GSK3β.^39–42^ Aberrant GSK3β expression and activity elicit the development of numerous diseases such as diabetes, cancer and AD.^43^ Therefore, GSK3β is considered an important target in drug discovery research and many inhibitory candidates are under characterization.^44–46^

The identification of small molecules affecting a target protein has been enhanced by the growing field of computational drug discovery. Computer-aided drug discovery involves a virtual investigation of the protein-ligand interactions and provides a theoretical basis for certain target inhibition.^47^ Furthermore, the drug discovery process has been accelerated by the newly introduced drug repurposing concept.^48^ Drug repurposing or repositioning offers a quick conveyance of clinically used medications to a different indication due to their well-proven safety and pharmacokinetics records.^49^ Accordingly, third-generation cephalosporin antibiotics are introduced herein to have the structural features required for GSK3β inhibition. Cephalosporins are among the most exploited family of antibiotics used for the treatment of bacterial infections.^16^ However, these β-lactam antibiotics proved more beneficial than just killing pathogenic bacteria. Several studies reported the benefits of β-lactam-containing compounds against diabetes, cancer and AD. β-Lactams showed antihyperglycemic effects as evidenced by decreased serum glucose and increased activity of glycogen synthase.^50,51^ Cephalosporins were reported to upregulate P53 apoptotic pathway leading to induced apoptosis and decreased proliferation of nasopharyngeal carcinoma cells.^19^ In AD, cephalosporins have been repeatedly noted to tame down neuropathological pathways, decreasing tau hyperphosphorylation and amyloid-β peptide production.^52,53^ GSK3β controls all of these pathophysiologic pathways implying that the benefits of cephalosporin antibiotics could be mediated by GSK3-β inhibition.^54,55^ Furthermore, it appears from the literature reports that third-generation cephalosporins have the most prominent effects against cancer and AD.^20^

In light of these observations, we docked the four cephalosporins, cefixime, ceftriaxone, cephalexin and cefadroxil against the active site of GSK3β. The highest-ranking poses of cefixime and ceftriaxone demonstrated the thiazole NH_2_ as a hydrogen bond donor, interacting with the carbonyl oxygens of Val-135 and Pro-136 in the hinge region. The two compounds showed opposite orthogonal binding orientations in agreement with the reported existence of at least two distinct binding modes accessible to ligands within the GSK3β active site.^56^ Cephalexin and cefadroxil lack the aminothiazole group and their top-ranked poses failed to interact with the hinge residues. Interactions within GSK3β binding pocket displayed by cefixime and ceftriaxone were proven stable during 100 ns MD simulations. Interestingly, the electrophilic carbonyl carbon of the β-lactam ring approached the nucleophilic trap of Cys-199, suggesting potential covalent bond formation. Covalent docking illustrated this interaction and resulted in binding modes consistent with those predicted by SP docking. To validate our computational approach, cefixime, ceftriaxone, cephalexin and cefadroxil were bioassayed *in vitro* against GSK3β. Cefixime and ceftriaxone showed the best inhibitory results with low micromolar IC_50_ values. To evaluate irreversible inhibition, cefixime and ceftriaxone were preincubated for 60 minutes with GSK3β before measuring their activity. Prolonged exposure to nucleophilic cysteines in kinases is necessary to ensure the formation of a covalent bond.^57^ This extended incubation period allows for a better alignment of the reactive electrophile and the nucleophilic trap after the formation of a reversible complex.^38^ Cefixime and ceftriaxone exhibited submicromolar IC_50_ values, with a 1-fold shift in the IC_50_ observed in the case of ceftriaxone. The increased potency after preincubation supports the formation of a covalent bond between the β-lactam carbonyl carbon and the thiol of Cys-199, as predicted by the MD simulations. This covalent bond results in a more stable binding to the kinase and a stronger inhibition of its activity.

According to Glide SP docking, the additional hydrogen bond between the thiazole sulfur and Val-135 NH in ceftriaxone, and the subsequent more negative interaction energy (−61.38 kcal mol^−1^), indicate a favorable potency profile compared to cefixime (interaction energy −48.28 kcal mol^−1^). However, both compounds were equipotent *in vitro*, with and without preincubation. This could be due to the more hydrophilic nature of ceftriaxone, as demonstrated by its lower log *P* value (−1.7) and higher topological polar surface area (288 Å^2^), compared to cefixime (log *P* = −0.4 and topological polar surface area = 238 Å^2^). The higher hydrophilicity increases the desolvation effect, leading to a greater entropic cost and, subsequently, lower affinity. Our docking protocol does not consider the entropic contribution of desolvation effect, and therefore, ceftriaxone showed a more negative interaction energy due to the enthalpically favorable interaction of the thiazole sulfur with Val-135 NH. Although this additional hydrogen bond can overcome the entropically unfavorable hydrophilicity of ceftriaxone, it was observed to rapidly detach in MD simulations as the β-lactam carbonyl moved closer to Cys-199 thiol.

In this study, we provided evidence for the mechanism by which cephalosporin antibiotics exert their beneficial effects against cancer and AD, namely, by inhibiting GSK3β. The hinge-interacting, aminothiazole group is common in third-generation cephalosporins, justifying the observed supremacy of this class in counteracting the effects of GSK3β downstream substrates in cancer and AD. Since cephalosporins are approved drugs with good safety profiles, this drug repurposing strategy represents a promising starting point. However, the ability of cephalosporins to cross the cell membrane and the blood–brain barrier is relatively poor due to their high polarity.^58^ This feature explains the high IC_50_ values of the third-generation cephalosporins, cefotaxime and ceftazidime, obtained by measuring cell viability of nasopharyngeal carcinoma cells after 72 hours of treatment (about 200 μM).^19^ Therefore, the elimination of the polar groups, particularly the solvent-exposed parts in cefixime and ceftriaxone, could enhance greater membrane permeability and consequently better activity.

## 5. Conclusion

Encouraged by the observed anticancer and anti-AD effects of cephalosporin antibiotics, we computationally assessed the potential of four cephalosporins to show GSK3β inhibitory activity. The third-generation compounds, cefixime and ceftriaxone, appeared promising and exerted micromolar inhibitory activities. Prolonged incubation of both compounds with GSK3β at different concentrations revealed covalent inhibition. Overall, our results introduce cephalosporins as GSK3β covalent inhibitors, providing evidence of the mechanism of action underlying their benefits in cancer and AD.

## Conflicts of interest

The authors declare no conflicts of interest.

## Supplementary Material

RA-013-D3RA01145C-s001
