# Supplementary material for: Molecular docking, molecular dynamics simulations and in vitro screening reveal cefixime and ceftriaxone as GSK3β covalent inhibitors
Source: RSC Adv. 2023 Apr 11;13(17):11278–90. doi: 10.1039/d3ra01145c (PMC10087387; doi:10.1039/d3ra01145c)
Supplement: RA-013-D3RA01145C-s001 [file RA-013-D3RA01145C-s001.pdf]

Electronic Supplementary Material (ESI) for RSC Advances.
This journal is © The Royal Society of Chemistry 2023

Supplementary information for:

Molecular Docking, Molecular Dynamics Simulations and In Vitro Screening Reveal Cefixime and Ceftriaxone as GSK3β Covalent Inhibitors.

Husam Nassar[1], Wolfgang Sippl[1], Rana Abu Dahab[2*], Mutasem Taha[3*]

[1]Department of Medicinal Chemistry, Institute of Pharmacy, Martin-Luther University Halle-Wittenberg, Halle (Saale) 06120, Germany

[2]Department of Clinical Pharmacy and Biopharmaceutics, School of Pharmacy, University of Jordan, Amman 11942, Jordan

[3]Department of Pharmaceutical Sciences, School of Pharmacy, University of Jordan, Amman 11942, Jordan

[*]To whom correspondence should be addressed.

Prof. Dr. Rana Abu Dahab

Telephone: +96265355000, extension 23353

Fax: +96265339649. Email: abudahab@ju.edu.jo

Prof. Dr. Mutasem Taha

Telephone: +96265355000, extension 23305.

Fax: +96265339649. E-mail: mutasem@ju.edu.jo.

- **Top-ranked poses of cephalexin and cefadroxil produced by Glide SP docking:**

  Clearly from the figure, Glide failed to predict any interaction with the hinge residues, confirming the essential role of the aminothiazole moiety in the activity and justifying the inferior inhibitory profile of cephalexin and cefadroxil as compared to cefixime and ceftriaxone.

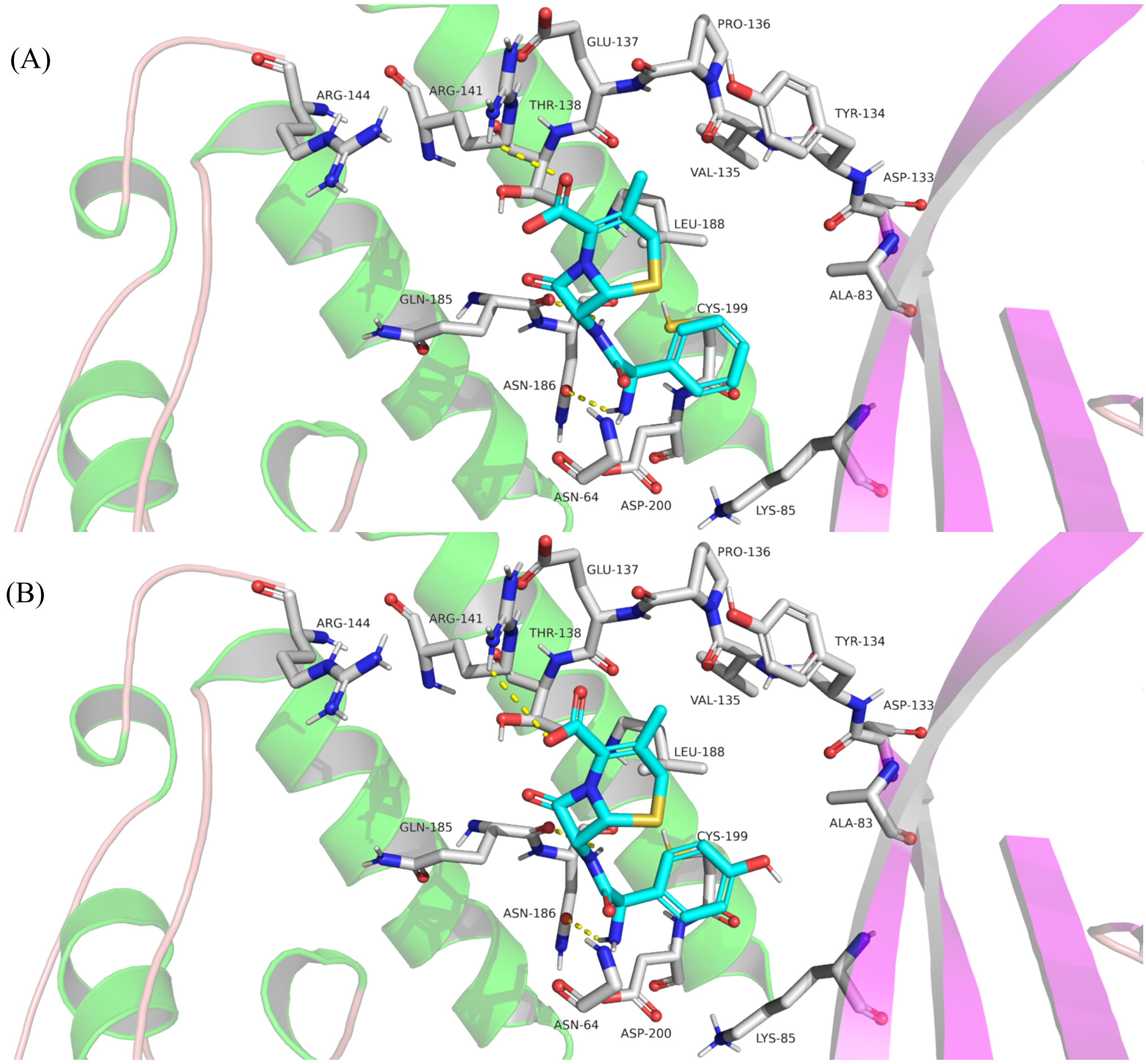

Fig. S1 Detailed view showing the binding modes of cephalexin (A) and cefadroxil (B) within the binding pocket of GSK3β. Hydrogen bonds (distance below 3.0 Å) are shown as yellow lines.

- **Other binding modes of cefixime and ceftriaxone produced by Glide SP docking:**

  The stability of the binding modes depicted in figure 3 was compared with the stability of the poses presented in this figure through 100 ns MD simulations.

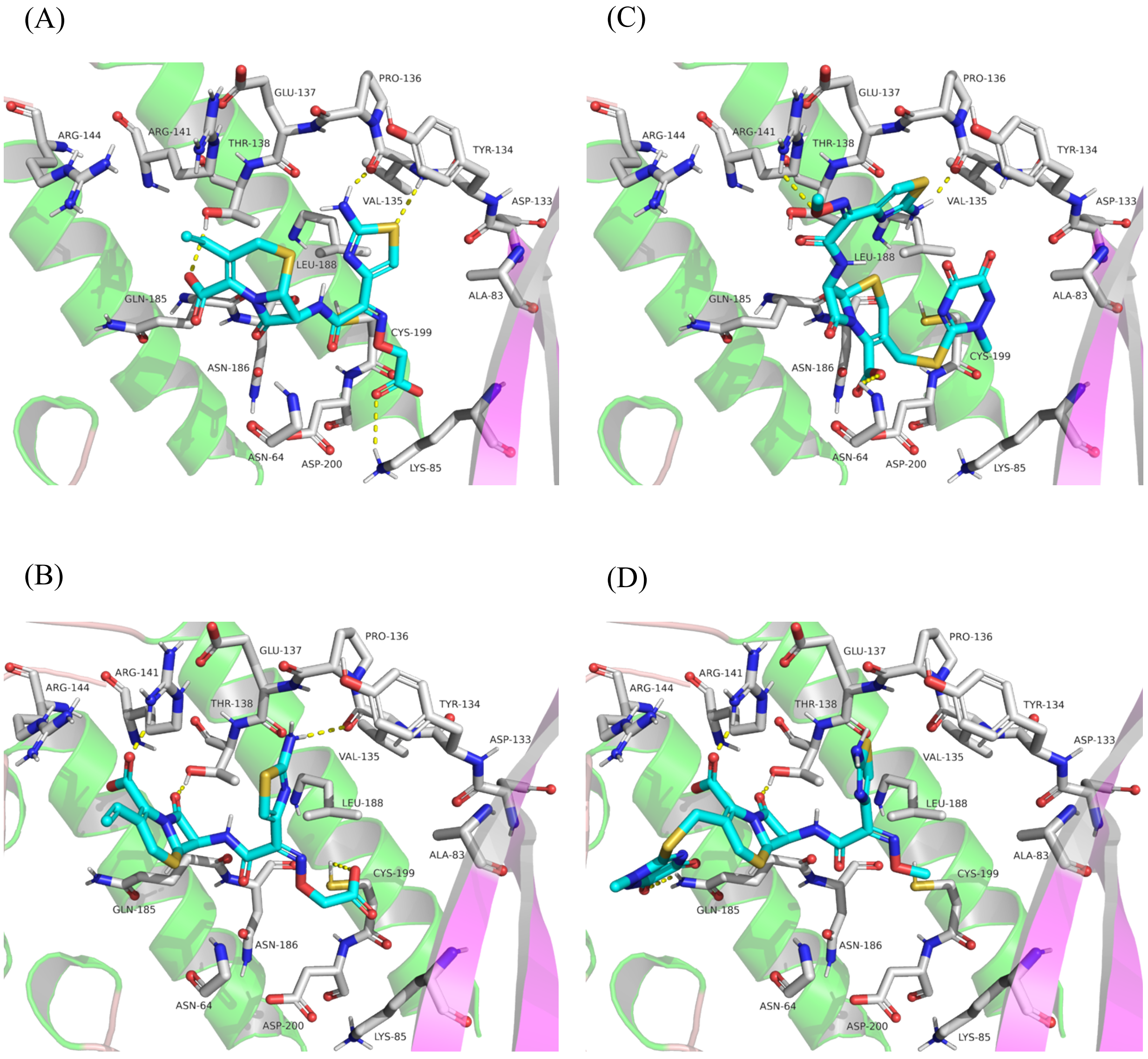

Fig. S2 Detailed view showing different binding modes of cefixime (A) and (B) and ceftriaxone (C) and (D) within the binding pocket of GSK3β. Hydrogen bonds (distance below 3.0 Å) are shown as yellow lines.

- **RMSD plots of complexes depicted in figure S2:**

RMSD measures the average change in displacement of ligand atoms for all frames with respect to the reference frame zero. The RMSD for frame x is:

$$\mathrm{RMSD_x} = \sqrt{\frac{1}{N}\sum_{i=0}^{n}(r_i'(t_x)) - r_i(t_{ref}))}\;{}_2$$

Where N is the number of atoms in the atom selection; t_{ref} is the reference time, (typically t=0); and r′ is the position of the selected atoms in frame x after superimposing on the reference frame, where frame x is recorded at time t_x. The procedure is repeated for every frame in the simulation trajectory. Clearly from the figure, RMSD values are high as compared to the top-ranked poses.

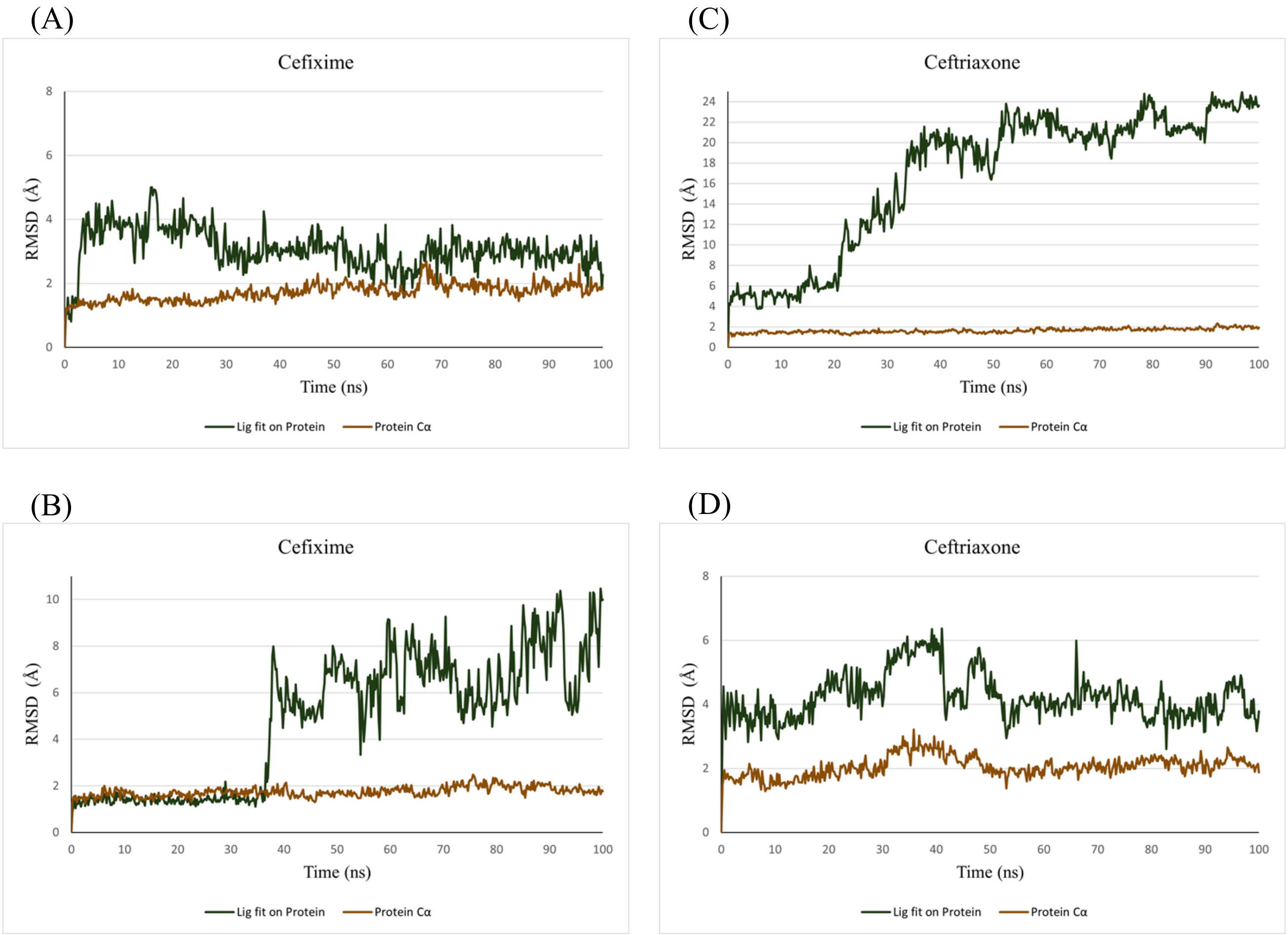

Fig. S3 RMSD values during 100 ns MD simulations of cefixime (A) and (B) and ceftriaxone (C) and (D) poses as presented in figure S2. RMSD values of protein Cα and docked poses fitting on protein Cα are represented in brown and green, respectively.

- **Analysis of the MD simulations replica of the top-ranked cefixime and ceftriaxone poses conducted at a different random seed:**

  To further confirm the stability of the binding modes and interactions of top-ranked poses, the MD simulations were repeated at different random seeds. The RMSD and RMSF values were consistent, confirming the observed stability in comparison to the other binding modes.

(A)

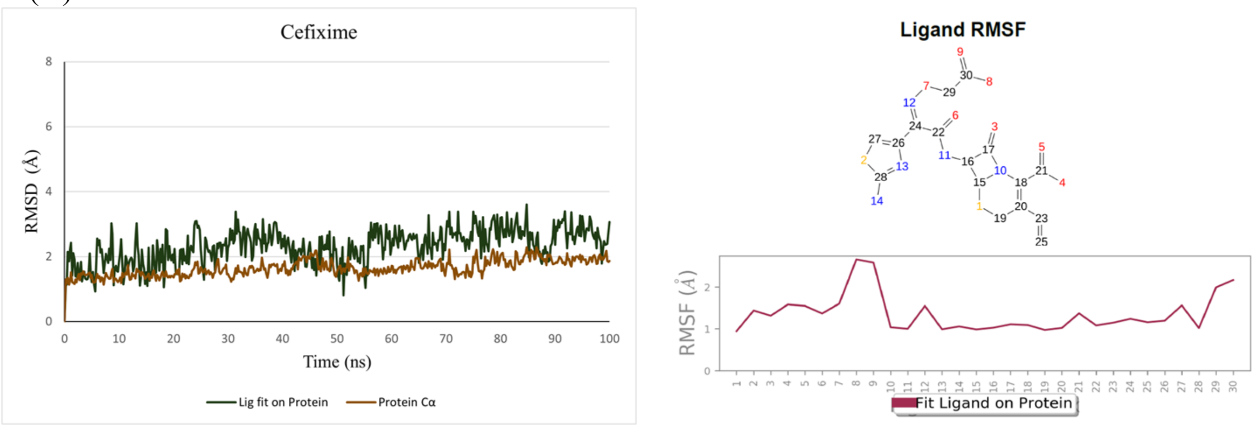

(B)

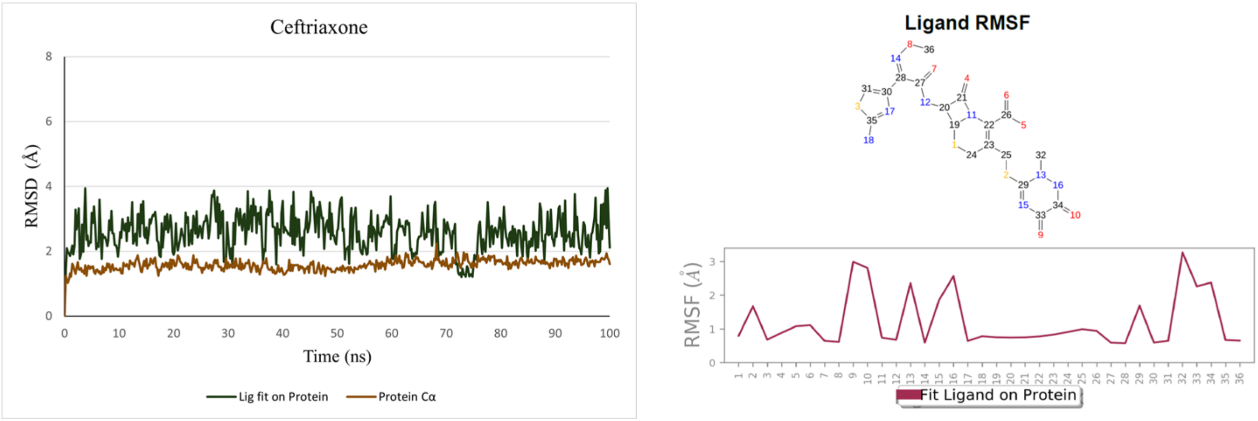

Fig. S4 Analysis of 100 ns MD simulations replica of the top-ranked poses of cefixime (A) and ceftriaxone (B). RMSD values of protein Cα and docked poses fitting on protein Cα are represented in brown and green, respectively. In the right are plotted the RMSF values broken down by atom.

- **Ligand-protein interaction fractions:**

Protein interactions with cefixime and ceftriaxone were monitored throughout the simulation. These interactions are categorized by type and summarized, as shown in the plots below. Protein-ligand interactions are categorized into four types: Hydrogen Bonds, Hydrophobic, Ionic and Water Bridges. The stacked bar charts are normalized over the course of the trajectory: for example, a value of 0.7 suggests that 70% of the

simulation time the specific interaction is maintained. Values over 1.0 are possible as some protein residue may make multiple contacts of the same subtype with the ligand.

(A)

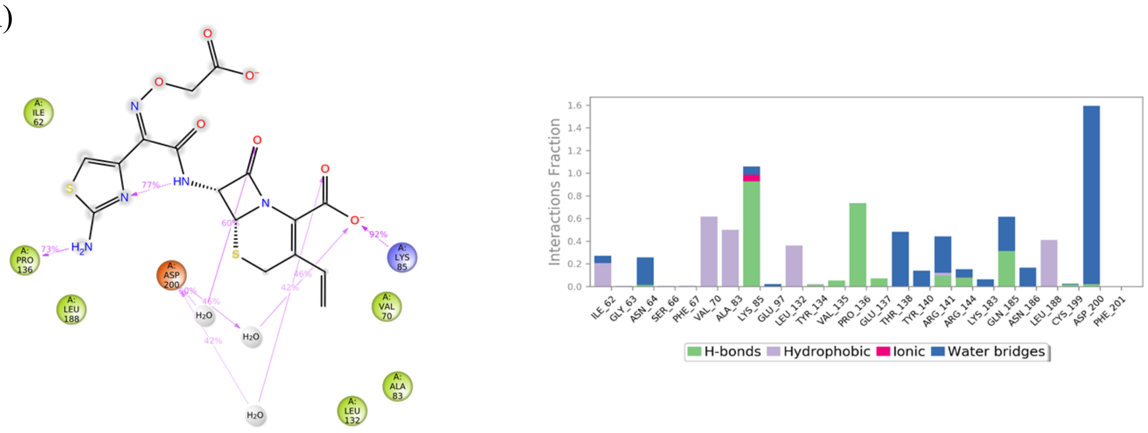

(B)

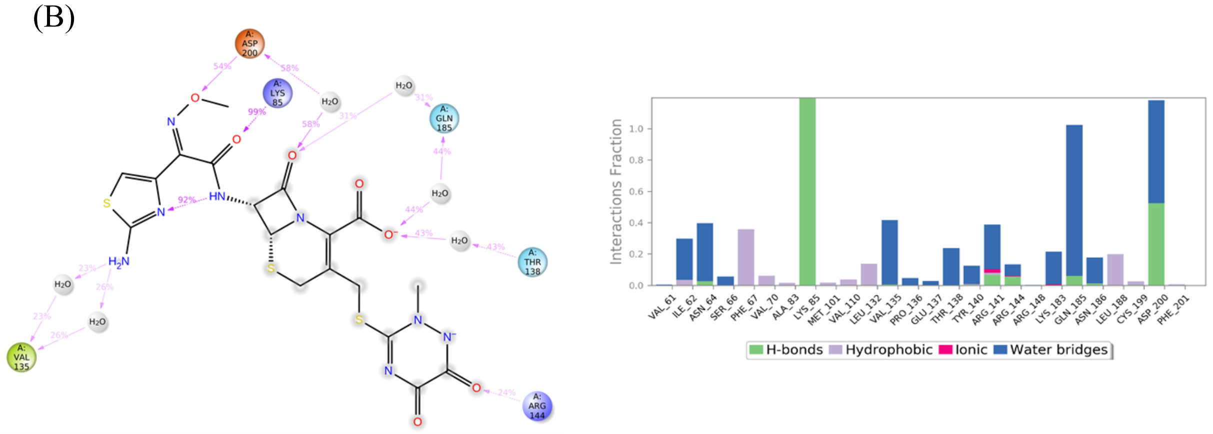

Fig. S5 A schematic of detailed cefixime (A) and ceftriaxone (B) interactions with the protein residues. In the right are summarized and categorized the protein interactions with each compound throughout the simulation. The geometric criteria for protein-ligand H-bond is: distance of 2.5 Å between the donor and acceptor atoms (D—H···A); a donor angle of ≥120° between the donor-hydrogen-acceptor atoms (D—H···A); and an acceptor angle of ≥90° between the hydrogen-acceptor-bonded_atom atoms (H···A—X).

- **Top-ranked poses of cephalexin and cefadroxil produced by covalent docking:**

(A) (B)

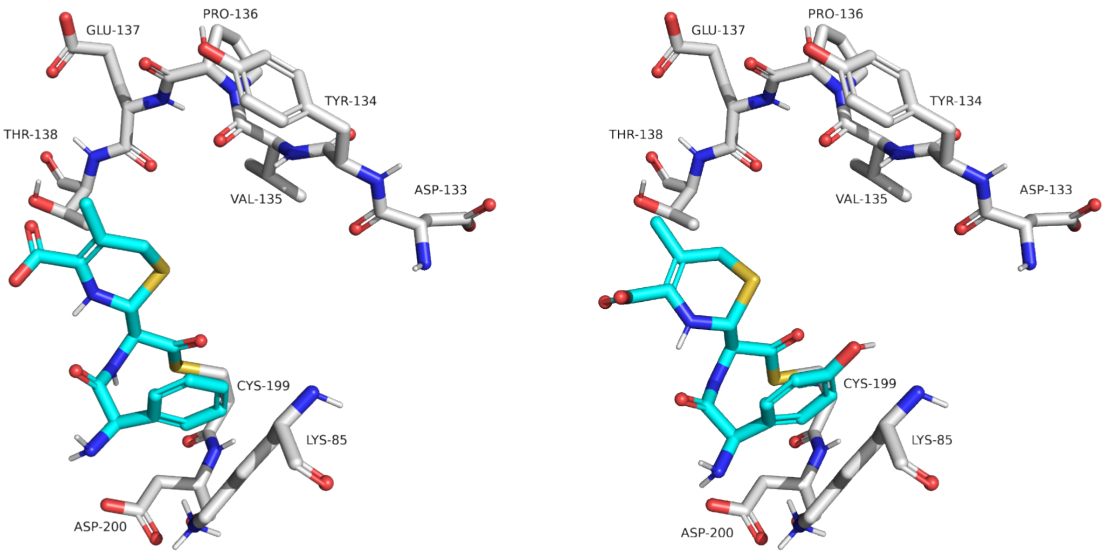

Fig. S6 Detailed view showing the predicted binding modes of Cephalexin (A) and cefadroxil (B) within the binding pocket of GSK3β upon covalent bond formation.

- **Analysis of the MD simulations of the covalently bound cefixime and ceftriaxone:**

To compare the stability and interaction patterns of cefixime and ceftriaxone before and after covalent bond formation, an additional MD simulations study was performed. The results were consistent as the complexes were stable and the interactions with hinge residues (Val-135 and Pro-136), catalytic Lys-85 and Asp-200 in the DFG motif were sustained upon covalent bond formation.

(A)

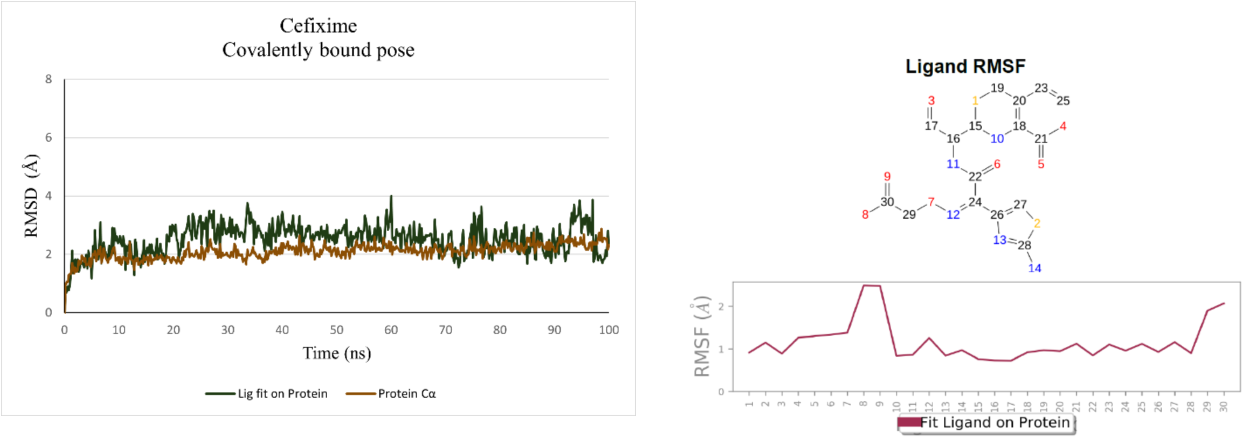

(B)

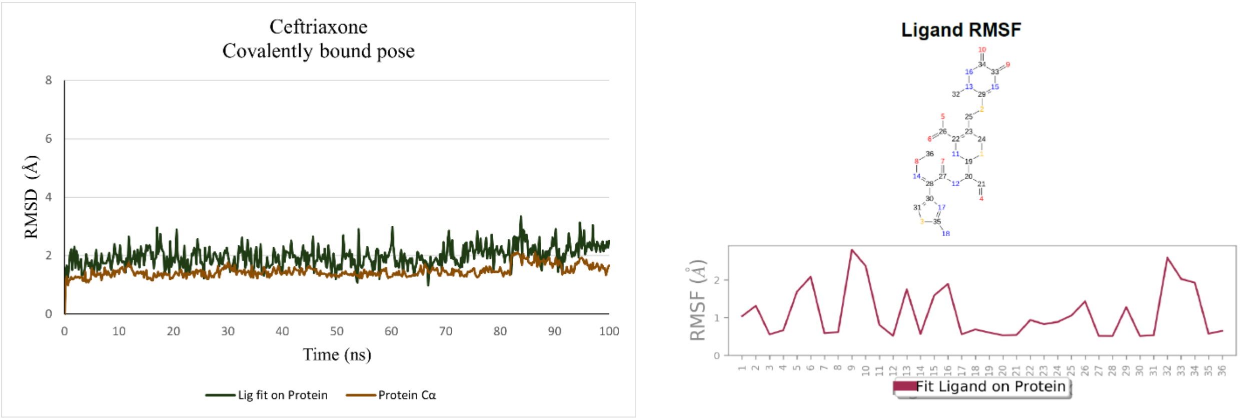

Fig. S7 Analysis of additional 100 ns MD simulations of the covalently bound poses of cefixime (A) and ceftriaxone (B). RMSD values of protein Cα and docked poses fitting on protein Cα are represented in brown and green, respectively. In the right are plotted the RMSF values broken down by atom.

- **Ligand-protein interaction fractions of the covalently bound complexes:**

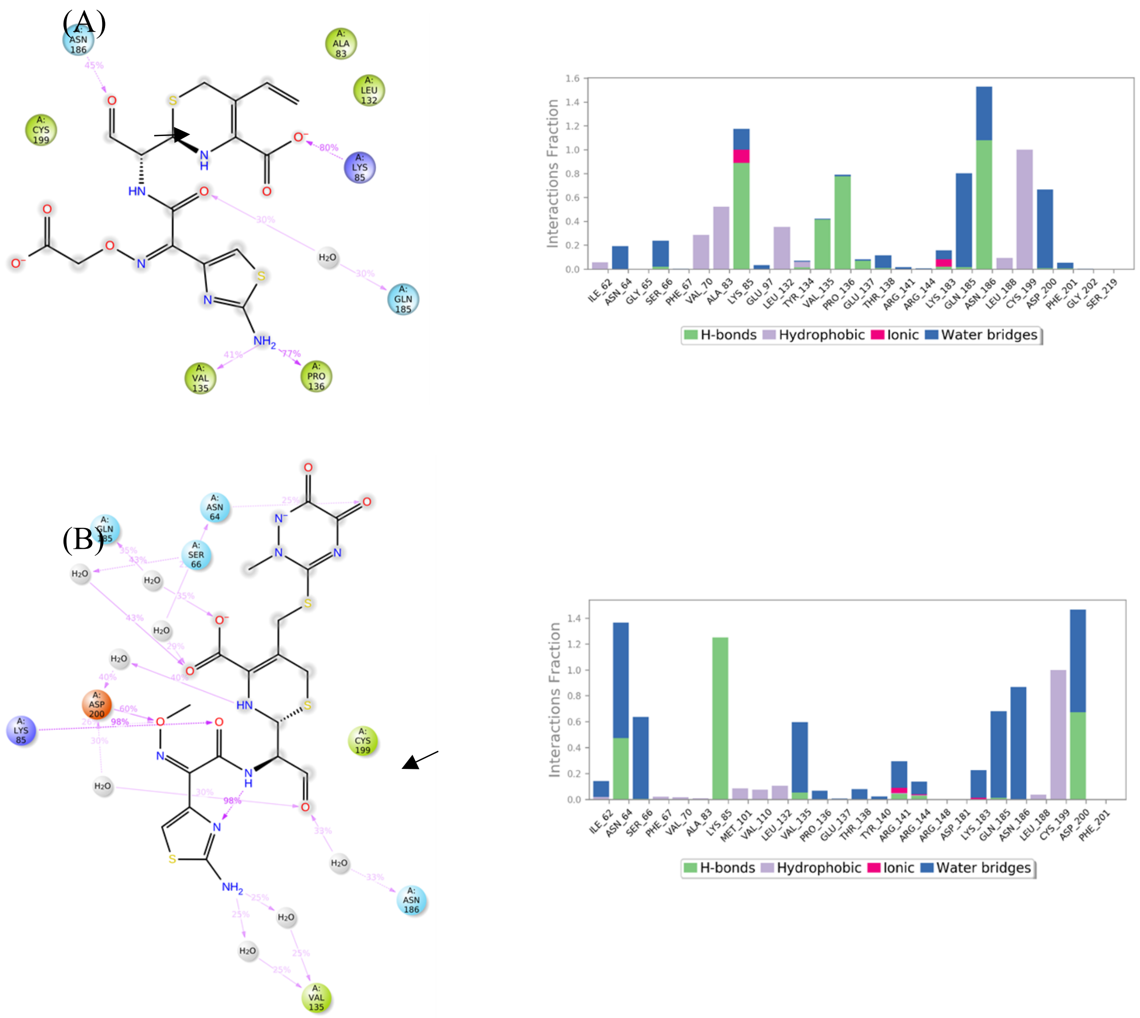

Fig. S8 A schematic of detailed the covalently bound cefixime (A) and ceftriaxone (B) interactions with the protein residues. In the right are summarized and categorized the protein interactions with each compound throughout the simulation. The geometric criteria for protein-ligand H-bond is: distance of 2.5 Å between the donor and acceptor atoms (D—H···A); a donor angle of ≥120° between the donor-hydrogen-acceptor atoms (D—H···A); and an acceptor angle of ≥90° between the hydrogen-acceptor-bonded_atom atoms (H···A—X). The black arrow indicates the site of the covalent bond.
